# Therapeutic Effects of Aripiprazole in the 5xFAD Alzheimer’s Disease Mouse Model

**DOI:** 10.3390/ijms22179374

**Published:** 2021-08-29

**Authors:** Ye Ji Jeong, Yeonghoon Son, Hye-Jin Park, Se Jong Oh, Jae Yong Choi, Young-Gyu Ko, Hae-June Lee

**Affiliations:** 1 Division of Radiation Bioscience, Korea Institute of Radiological & Medical Sciences, Seoul 01812, Korea; whyj0914@kirams.re.kr (Y.J.J.); sonyh@kirams.re.kr (Y.S.); jhp13@hanmail.net (H.-J.P.); 2 Division of Life Sciences, Korea University, Seoul 02841, Korea; ygko@korea.ac.kr; 3 Division of Applied RI, Korea Institute of Radiological and Medical Sciences, Seoul 01812, Korea; osj5353@kirams.re.kr (S.J.O.); smhany@kirams.re.kr (J.Y.C.)

**Keywords:** aripiprazole, Alzheimer’s disease mice, βA pathology, FDG-PET, therapeutic agent

## Abstract

Global aging has led to growing health concerns posed by Alzheimer’s disease (AD), the most common type of dementia. Aripiprazole is an atypical FDA-approved anti-psychotic drug with potential against AD. To investigate its therapeutic effects on AD pathology, we administered aripiprazole to 5xFAD AD model mice and examined beta-amyloid (βA)-induced AD-like phenotypes, including βA production, neuroinflammation, and cerebral glucose metabolism. Aripiprazole administration significantly decreased βA accumulation in the brains of 5xFAD AD mice. Aripiprazole significantly modified amyloid precursor protein processing, including carboxyl-terminal fragment β and βA, a disintegrin and metalloproteinase domain-containing protein 10, and beta-site APP cleaving enzyme 1, as determined by Western blotting. Neuroinflammation, as evidenced by ionized calcium binding adapter molecule 1 and glial fibrillary acidic protein upregulation was dramatically inhibited, and the neuron cell layer of the hippocampal CA1 region was preserved following aripiprazole administration. In 18F-fluorodeoxyglucose positron emission tomography, after receiving aripiprazole, 5xFAD mice showed a significant increase in glucose uptake in the striatum, thalamus, and hippocampus compared to vehicle-treated AD mice. Thus, aripiprazole effectively alleviated βA lesions and prevented the decline of cerebral glucose metabolism in 5xFAD AD mice, suggesting its potential for βA metabolic modification and highlighting its therapeutic effect over AD progression.

## 1. Introduction

In 2018, an estimated 50 million people worldwide had Alzheimer’s dementia, a number expected to increase to >152 million by 2050 [1]. Progressive population aging has led to a rapid increase in the incidence of Alzheimer’s disease (AD), and a growing need for an effective therapeutic drug against it. AD is the most common dementia and progressive neurodegenerative disease. Its core pathological substrates in the AD brain are amyloid plaques and neurofibrillary tangles [2] and there are other potential mechanisms of AD pathophysiology, including neuroinflammation, protein misfolding, mitochondrial dysfunction, and clearance of abnormal proteins [3]. In particular, increased production of beta-amyloid (βA) species, whose aggregation and deposition as insoluble plaques is regarded as an early and key pathological marker in the development of AD [4]. Despite compelling genetic and molecular evidence pointing to βA as a key player in AD pathogenesis [5], the majority of clinical trials targeting βA cascades have produced negative results [6,7]. However, amyloid remains the most compelling therapeutic target [8] and efforts to find novel therapeutics for AD using new technologies, including in silico drug repurposing [9], or FDA-approved drugs [10] are ongoing.

Aripiprazole is a second-generation atypical antipsychotic with high affinity for D2, D3, 2-HT1a, and 5-HT2a receptors, and moderate affinity for D4, 5-HT2c, 5-HT7, alpha 1 adrenergic, and H1 receptors [11]. Aripiprazole is approved for use in patients with various psychotic disorders, including schizophrenia, bipolar disorder, depression, and autism [12,13,14], and it has been reported that some clinical studies showed beneficial effect of aripiprazole for AD-related psychosis, such as aggressiveness or anxiousness [15,16]. In addition to its effects on psychiatric disorders, the neuroprotective effect of aripiprazole on trimethyltin-induced neuron loss [16] or traumatic brain injury [17] has been reported in rodents. Recently, aripiprazole was proposed as a therapeutic agent for AD by an in silico deep machine learning analysis of FDA-approved drugs [10]. However, the therapeutic effects of aripiprazole on progressive AD and its modulatory effects over the βA pathway have not been investigated and animal experiments would help to shed light on the therapeutic mechanisms involved.

Therefore, in this study, we assessed the impact of aripiprazole on AD progression using the 5xFAD AD model mouse at five months of age when βA plaques are deposited throughout the hippocampus in this model. We performed histological and molecular analyses targeting βA-induced AD-like phenotypes in the brain, including amyloid precursor protein (APP) cleavage and cleaving enzymes, immune activation, and neuronal damage. We also examined the effect of aripiprazole on cerebral glucose metabolism dysfunction in AD mice.

## 2. Results

### 2.1. Aripiprazole Effectively Inhibited βA Deposition in the Brain of 5xFAD AD Mice

**Figure 1:**
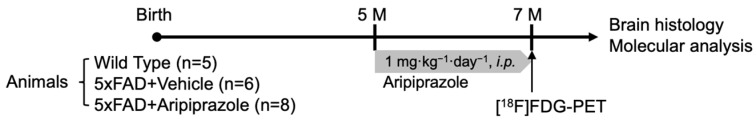
Illustration of experimental design. At the age of 5 months, aripiprazole (1 mg·kg^−1^·day^−1^) or vehicle were intraperitoneally administered to age-matched wild-type (WT) and 5xFAD for 2 months. At the age of 7 months, animals underwent 18F-fluorodeoxyglucose positron emission tomography ([^18^F]FDG-PET) image and were then sacrificed for further histopathological and molecular analyses.

**Figure 2:**
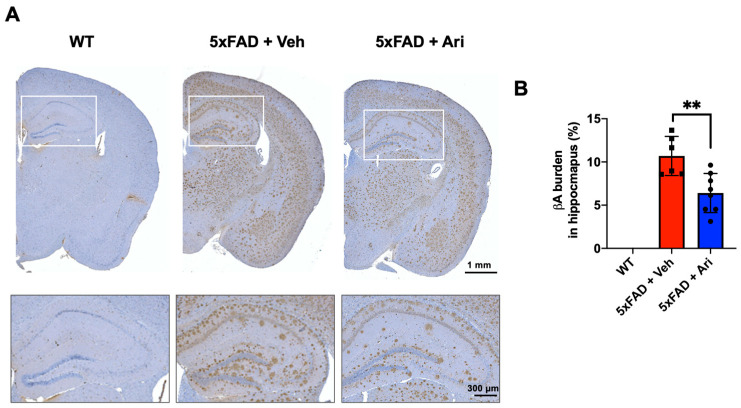
Aripiprazole administration decreased β-amyloid deposition in the hippocampus of 5xFAD mice. (**A**) Representative immunohistochemistry images of βA deposits (in brown) in the brain of 5xFAD mice treated with aripiprazole. Squares show higher magnification of hippocampus regions. (**B**) Quantification of βA burden in the hippocampus of WT (*n* = 5), 5xFAD + Veh (*n* = 6), and 5xFAD + Ari (*n* = 7) group. 5xFAD + Ari and 5xFAD + Veh groups showed statistically significant difference in βA burden (Student’s two-tailed *t*-test, ** *p* < 0.01). Error bars indicate SD.

To investigate the effect of aripiprazole in a mouse model of AD, 5xFAD mice at the age of 5 months were treated with aripiprazole (Ari) by intraperitoneal injection at 1 mg·kg^−1^ for 2 months (Figure 1). Treatment with aripiprazole effectively attenuated βA accumulation in the brain parenchyma compared to vehicle-treated 5xFAD controls (5xFAD + Veh). The βA burden in the hippocampus was significantly reduced—up to 40%—by aripiprazole administration in 5xFAD AD mice (Student’s two-tailed *t*-test, *p* = 0.004, 5xFAD + Veh = 10.69 ± 2.36 vs. 5xFAD + Ari = 6.41 ± 2.26, Figure 2).

**Figure 3:**
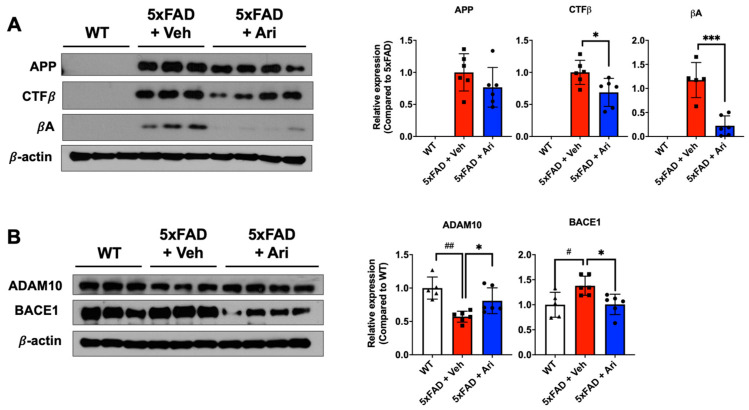
Aripiprazole effectively inhibited βA cascades in 5xFAD mice. (**A**) Representative Western blot images and APP, CTFβ, and βA quantification in the hippocampus of the indicated groups. Statistical significance using Student’s two-tailed *t*-test defined as * *p* < 0.05, *** *p* < 0.001 5xFAD + Veh (*n* = 6) vs 5xFAD + Ari (*n* = 8) group; error bars indicate SD. (**B**) Representative Western blot images and quantification of expression levels of APP cleaving enzymes ADAM10 and BACE1. Statistical significance using one-way ANOVA defined as ^#^ *p* < 0.05, ^##^ *p* < 0.01 5xFAD + Veh (*n* = 6) vs. WT (*n* = 5) group; * *p* < 0.05, *** *p* < 0.001 5xFAD + Veh vs. 5xFAD + Ari (*n* = 6) group; error bars indicate SD.

To investigate the effect of aripiprazole on βA production, we performed Western blot analysis of hippocampal samples from each group to determine the expression levels of APP metabolites, including APP, carboxyl-terminal fragment β (CTFβ), and βA. In addition, the protein levels of their cleavage enzymes, including a disintegrin and metalloproteinase domain-containing protein 10 (ADAM10) and beta-site APP cleaving enzyme (BACE1), were evaluated. Consistent with the immunohistochemistry results, the brains of the WT group showed no protein expression of APP, CTFβ, and βA, while those of 5xFAD + Veh mice showed high expression of all of them. Further, aripiprazole administration effectively suppressed APP metabolites, 31% CTFβ expression (5xFAD + Ari = 0.69 ± 0.22, Student’s two-tailed *t*-test *p* = 0.024) and 73% βA expression (5xFAD + Ari = 0.22 ± 0.21, *p* = 0.0004) compared to 5xFAD + Veh group (Figure 3A). 5xFAD + Veh mice showed lower ADAM10 expression (5xFAD + Veh = 0.57 ± 0.08, one-way ANOVA analysis of variance, *p* = 0.001) and higher BACE1 expression (5xFAD + Veh = 1.38 ± 0.19, *p* = 0.028) than WT mice. Following aripiprazole treatment, the levels of APP cleaving enzymes, BACE1 (5xFAD + Ari = 1.01 ± 0.02, one-way ANOVA *p* = 0.024, 5xFAD + Veh vs 5xFAD + Ari) and ADAM10 (5xFAD + Ari = 0.81 ± 0.19, *p* = 0.044, 5xFAD + Veh vs. 5xFAD + Ari) in the hippocampus recovered to near-WT levels (Figure 3B).

### 2.2. Aripiprazole Suppressed Neuroinflammation in 5xFAD AD Mice

**Figure 4:**
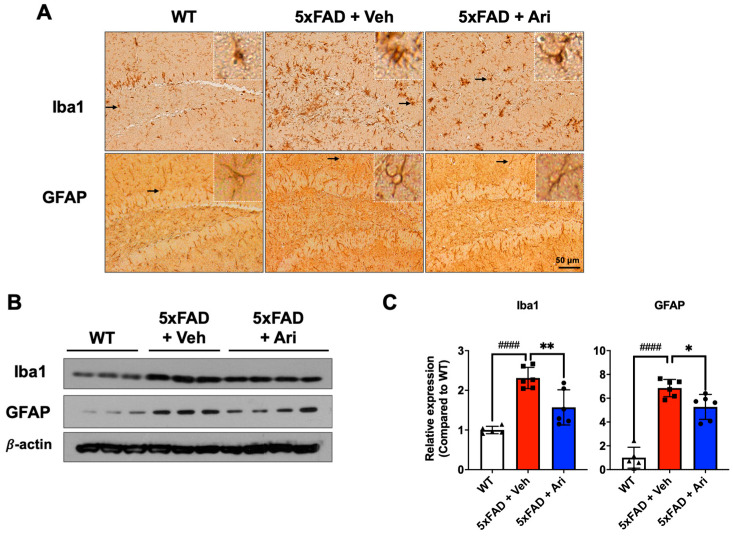
Aripiprazole treatment attenuated neuroinflammation in the hippocampus of 5xFAD mice. (**A**) Representative immunohistochemistry images of active microglia (Iba1-positive) and astrocytes (GFAP-positive) in 5xFAD mice treated with aripiprazole. Brown indicates Iba1 or GFAP positive staining. Inserted squares represent high magnification images of microglia or astrocytes as indicated with an arrow. Scale bar = 50 μm. (**B**) Representative Western blots of Iba1 and GFAP in the hippocampus of WT, 5xFAD + Veh and 5xFAD + Ari mice. (**C**) Quantification of Iba1 and GFAP expression in hippocampal lysates for the indicated groups. Statistical significance using one-way ANOVA defined as ^####^ *p* < 0.001, WT (*n* = 5) vs. 5xFAD + Veh (*n* = 6); * *p* < 0.05, ** *p* < 0.01, 5xFAD + Veh vs. 5xFAD + Ari (*n* = 6) group. Error bars indicate SD.

5xFAD AD mice showed significant activation of immune cells, as shown by microglia and astrocyte activation (Figure 4A). Western blotting and quantification revealed that 5xFAD + Veh mice had significantly higher levels of ionized calcium-binding adapter molecule 1 (Iba1) (5xFAD + Veh = 2.31 ± 0.27, one-way ANOVA *p* < 0.0001) and glial fibrillary acidic protein (GFAP) (5xFAD + Veh = 6.85 ± 0.72, *p* < 0.0001) than the WT group. Aripiprazole treatment led to significant Iba1 (5xFAD + Ari = 1.57 ± 0.44, *p* = 0.003, 5xFAD + Veh vs. 5xFAD + Ari) and GFAP (5xFAD + Ari = 5.27 ± 1.05, *p* = 0.022, 5xFAD + Veh vs. 5xFAD + Ari) suppression in the hippocampus (Figure 4B,C).

### 2.3. Aripiprazole Prevented Neuronal Loss in the Hippocampus of 5xFAD Mice

**Figure 5:**
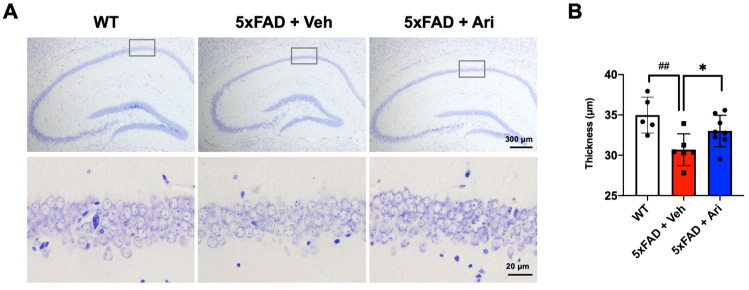
Aripiprazole prevented neuron loss in the hippocampus of 5xFAD mice. (**A**) Representative cresyl violet staining images of the hippocampal region of the indicated groups and (**B**) thickness quantification in the CA1 region. Statistical significance using one-way ANOVA defined as ^##^ *p* < 0.01 WT (*n* = 5) vs. 5xFAD + Veh (*n* = 6); * *p* < 0.05 5xFAD + Veh vs. 5xFAD + Ari (*n* = 8) group. Error bars indicate SD.

Since we observed that aripiprazole dramatically lowered the hippocampal βA load, we next investigated the neuronal population representing the cell layer thickness of the CA1 region. In 5xFAD + Veh mice, the CA1 thickness (5xFAD + Veh = 35.19 ± 2.52, one-way ANOVA *p* = 0.028, WT vs. 5xFAD + Veh) was significantly lower than in WT mice (WT = 30.77 ± 2.19). Aripiprazole protected against neuron loss in the hippocampus (5xFAD + Ari = 33.04 ± 2.10, *p* < 0.01, compared to 5xFAD + Veh, Figure 5).

### 2.4. Aripiprazole Effectively Prevented the Decline of Cerebral Glucose Metabolism in 5xFAD Mice

To assess cerebral glucose metabolism, we performed [^18^F]FDG PET scans. A comparative overview of average [^18^F]FDG PET images (40–60 min p.i.) is shown in Figure 6A. The radioactivity of cortical and subcortical areas in the 5xFAD + Veh group displayed lower uptake than the WT group. However, after aripiprazole administration (5xFAD + Ari), the brain uptake dramatically improved. In terms of SUV (standardized uptake value), the uptake of target regions in the 5xFAD + Veh group was 8–16% lower than in the WT group (Figure 6B and Table 1). The brain uptake values were 9–25% higher in the 5xFAD + Ari group than in the 5xFAD + Veh group. Among the target regions, the striatum (*p* = 0.03), thalamus (*p* = 0.01), and hippocampus (*p* = 0.004) showed statistically significant differences between 5xFAD + Veh and 5xFAD + Ari (Figure 6).

## 3. Discussion

In this study, we investigated the potential of aripiprazole as a new therapeutic option for βA-based AD pathology. After chronic aripiprazole administration to the 5xFAD AD mouse model, aripiprazole effectively inhibited βA plaque deposition in the hippocampus and suppressed βA signaling cascades, as shown by CTFβ and βA and downregulation of the βA-cleaving enzyme, BACE1. Consistent with the reduction of βA plaques in the brains of 5xFAD mice, activated microglia and astrocytes were markedly suppressed. Furthermore, after aripiprazole treatment, the decreased glucose metabolism was improved in various brain regions, including the hippocampus. In this study, we demonstrated the therapeutic effects of aripiprazole on progressive AD pathology via inhibition of βA signaling cascades.

Previous studies have also suggested the potential of aripiprazole as an AD therapeutic in preclinical models. Yoneyama et al. reported that aripiprazole (3 mg·kg^−1^ daily *i.p.* for 2 weeks) enhanced regeneration after trimethyltin-induced neuron loss [16]. Moreover, Besagar et al. showed that sub-chronic aripiprazole administration (0.1 mg·kg^−1^ daily *i.p.* for 18 days) after traumatic brain injury enhanced recovery [17]. However, aripiprazole (3 and 6 mg·kg^−1^) did not show any protective effect on MK-801-induced olfactory memory impairment [18]. Despite the reported beneficial effect of aripiprazole on AD-related psychosis, there are no reports on its therapeutic effects on βA pathology. Since many patients are diagnosed with AD when lesions are quite advanced, we examined the therapeutic effects on animals with moderate AD progression. We started drug administration at 5 months of age when the brains of 5xFAD mice displayed substantial βA plaques and activated neuroinflammation [19,20,21]. The drug dose administered in this study, 1 mg·kg^−1^, was based on literature review. Previous studies reported neuroprotective effects of aripiprazole using 0.1 mg·kg^−1^ and 3 mg·kg^−1^ within 18 days [16,17]. However, Picada et al. reported that injection of aripiprazole 1, 3, and 10 mg·kg^−1^ (daily *i.p.*) for 5 consecutive days showed decreased motor activity in the open field task [22]. In addition, we chose 1 mg·kg^−1^ of aripiprazole for the chronic administration because 5xFAD has aggressive and progressive βA pathology. Different from the result of Picada et al., chronic aripiprazole administration (1 mg·kg^−1^ for 2 months) did not induce impairment of motor activities in the open field test (data not shown). Chronic treatment with aripiprazole markedly reduced βA plaques in the brain (Figure 2), and subsequently, Western blotting demonstrated that APP cleaving CTFβ and βA, and cleaving enzyme BACE1 expression were decreased (Figure 3). Simultaneously, α-secretase ADAM10, an enzyme that inhibits βA peptide production via non-amyloidogenic APP cleavage [21], was significantly upregulated (Figure 3B). Similarly, Heo et al. showed that combination treatment with two (aripiprazole and cilostazole) or three (aripiprazole, cilostazole, and donepezil) drugs increased ADAM10 expression in N2a Swe cells via the SIRT1 pathway, while donepezil alone did not alter the ADAM10 expression [23]. These results suggest that aripiprazole modulates βA signaling cascades. However, in this study, we did not investigate the dose-dependent effect of aripiprazole. Therefore, additional experiments should be performed with low and higher doses of aripiprazole, as well as consider the non-linear and threshold effects.

We also found that aripiprazole alleviated severe neuroinflammation in 5xFAD mice at 7 months of age, which is the point when drug administration was terminated. Aripiprazole significantly suppressed both Iba1 and GFAP levels in 5xFAD mice compared to vehicle-treated mice based on Western blot analysis, consistent with the expression of Iba1 and GFAP in the hippocampal regions observed on immunohistochemistry (Figure 4). In addition, 5xFAD mice with aripiprazole treatment exhibited reactive astrocytosis and activated microglia in the hippocampus that correlated with βA plaque compared to vehicle-treated 5xFAD mice. Consistent with our results, some in vitro studies showed an inhibitory effect of aripiprazole on microglial activation via dopamine D(2) receptor-independent suppression of IFN-gamma-induced Ca^2+^ elevation in microglia [24] or transient receptor potential in melastatin 7 (TRPM7) channels [25]. Further studies are needed to clarify the detailed mechanisms underlying the βA signaling cascade and neuroinflammation.

**Figure 6:**
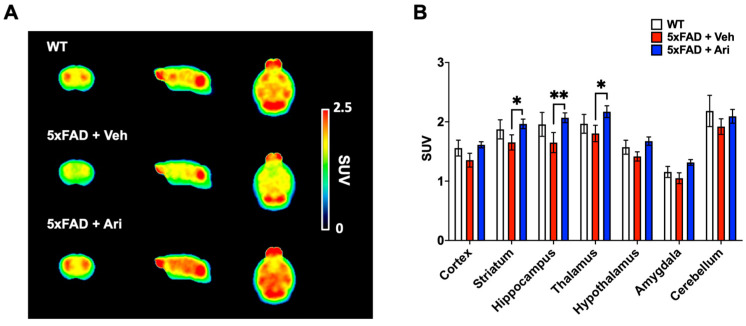
[^18^F]FDG-PET imaging for evaluating cerebral glucose metabolism following aripiprazole treatment. (**A**) Averaged [^18^F]FDG PET images between 40 and 60 min after injection in WT controls (*n* = 5), 5xFAD + Veh (*n* = 6) and 5xFAD + Ari mice (*n* = 8). Images are scaled to the SUV. (**B**) Quantification of SUV values for the target regions. Data are presented as mean ± SEM. * *p* < 0.05 and ** *p* < 0.01.

We confirmed the therapeutic effect of aripiprazole on AD mice using FDG-PET imaging. In patients, regional hypometabolism can predict cognitive decline and conversion to AD in persons with mild cognitive impairment [26,27], and distinguish AD from other forms of dementia and brain diseases [28]. Studies using transgenic mouse models of AD have also shown an association of metabolic dysfunction with neuroinflammation, and that both are early pathological features of AD, emerging before βA deposition [29,30,31]. In this study, compared to the vehicle-treated group, aripiprazole administration significantly improved the decline in cerebral glucose metabolism not only in the hippocampus but also in the striatum and thalamus (Figure 6). This result suggests its potential for functional improvements in the AD brain. Therefore, our findings are the first to demonstrate aripiprazole’s in vivo therapeutic effects on βA-based AD pathology using a 5xFAD mouse model. We also examined behavioral tests for memory task. Following aripiprazole administration, 5xFAD tended to improve memory function in Y-maze and novel object recognition tests, but the results did not reach statistical significance (data not shown). Because we used only 5–6 animals per WT and 5xFAD + Veh for the behavioral tests, this might be the reason for the lack of statistical significance. Therefore, additional experiments with larger sample sizes will be necessary to more accurately interpret behavioral changes in response to aripiprazole administration in 5xFAD mice. In addition, further studies are warranted to verify the functional brain changes in animal models of AD by PET scan and the associated behavioral functions following aripiprazole administration.

In conclusion, the current study demonstrated that aripiprazole, an FDA-approved antipsychotic drug, prevented βA deposition and neuroinflammation, major key AD pathological markers in progressive AD models. The therapeutic effects of aripiprazole on βA pathology were consistent with an improvement in cerebral glucose metabolism. Our findings may support aripiprazole as a potential disease-modifying therapy for AD.

## 4. Materials and Methods

### 4.1. Animals and Drug Administration

We used transgenic female 5xFAD mice with five mutant human genes associated with AD. 5xFAD mice express five familial forms of AD mutations on APP and presenilin-1 (PSEN1), three mutations in the APP gene (APP KM670/671NL (Swedish), APP I716V (Florida), APP V717I (London)), and two mutations in PSEN1 (PSEN1 M146L (A > C), PSEN1 L286V). 5xFAD mice show intraneuronal Aβ42 protein at 1.5 month of age, extracellular plaques at 2 months of age, and neuronal loss at 9 months of age. The method used for generating 5xFAD mice has been described previously [32]. Heterozygous 5xFAD transgenic animals and WT controls were obtained after breeding progenitors purchased from the Jackson Laboratory (Jackson Laboratory, Bar Harbor, ME, USA).

At 5 months of age, female 5xFAD mice were assigned to a vehicle control group (5xFAD + Veh: *n* = 6) or an aripiprazole-treated group (5xFAD + Ari: *n* = 8). Age-matched female B6/SJL mice were assigned to the wild-type group (WT: *n* = 5). As the pattern of beta amyloid accumulation and neuroinflammation is more severe in female 5xFAD mice than in males [33,34], we used only female 5xFAD and age-matched WT mice in our study. Aripiprazole (A2496; Tokyo Chemical Industry, Tokyo, Japan) was dissolved in dimethyl sulfoxide (DMSO) and further diluted in an aqueous mixture of 0.5% hydroxypropyl methyl cellulose (HPMC; Sigma-Aldrich, St. Louis, MO, USA) and 1% Tween 80 (Sigma-Aldrich). When the 5xFAD mice were 5 months old, aripiprazole was intraperitoneally administered to the mice at 1 mg·kg^−1^ (Figure 1). The mice were housed in a specific pathogen-free facility controlled at 22 ± 2 °C temperature and 60 ± 5% humidity with a 12:12 h light/dark cycle with access to a normal diet and autoclaved water *ad libitum*. All animal procedures in this study were approved by the Institutional Animal Care and Use Committee of the Korea Institute of Radiological and Medical Sciences (IACUC permit number: KIRAMS2019-0029, approval date: 17 May 2019).

### 4.2. Immunohistochemistry (IHC)

Tissue sections were deparaffinized in xylene and rehydrated in a gradient series of ethanol. The sections were heated in a citrate buffer (pH 6.0) in boiling water for 30 min for antigen retrieval. For blocking nonspecific staining, the slides were incubated with 1.5% normal horse serum (Vector Laboratories Inc., Burlingame, CA, USA) for 30 min, and then incubated with the following primary antibodies overnight at 4 °C: mouse anti-β-amyloid (6E10, 1:2000, sig-39320, Covance, Princeton, NJ, USA), anti-GFAP (1:1000, z0334, Dako, Carpinteria, CA, USA), and anti- Iba1 (1:1000, 019-19741, Wako, Osaka, Japan) antibodies. Subsequently, the slides were washed with 0.1% TritonX-100 PBS-T and incubated with biotinylated secondary antibodies (Vector Laboratories Inc.) for 30 min at room temperature. Slides were then incubated with an avidin–biotin–peroxidase complex (Vector Laboratories Inc.) for 30 min at room temperature. After incubation with diaminobenzidine (DAB, Vector Laboratories Inc.), images were obtained at 20× magnification using whole slide digital scanning with a digital pathology scanner (Aperio VERSA; Leica Biosystems, Richmond, IL, USA).

### 4.3. Cresyl Violet Staining

Brain sections were deparaffinized, rehydrated, and stained with 0.1% cresyl violet (C5042, Sigma-Aldrich, St. Louis, MO, USA) solution for 10 min at room temperature. After washing with distilled water, the slides were differentiated in 0.25% *v/v* glacial acetic acid in 95% ethyl alcohol for 10 s. Then, tissues were dehydrated in a gradient of alcohol, cleared in xylene, and mounted with coverslips. Slides were imaged using a light microscope (BX53; Olympus, Tokyo, Japan). The thickness of the CA1 region of the hippocampus was quantified by two blinded observers.

### 4.4. Western Blotting

The hippocampi were homogenized in a pro-prep solution (17081, iNtRon, Gyeonggi-do, Korea) after physically mincing. The protein concentration was quantified using the Bradford reagent (Bio-Rad, Hercules, CA, USA), and then denatured by boiling for 5 min, depending on the concentration of the sample. Each sample was separated by electrophoresis on 10–15% sodium dodecyl sulfate-polyacrylamide gels. Then, the proteins were transferred to a nitrocellulose membrane and blocked for 30 min with 5% bovine serum albumin (BSA) solution for nonspecific blocking. Membranes were incubated at 4 °C with anti-6E10 (1:1000, Sig-39320, Covance), anti-ADAM10 (1:1000, ab1997, Abcam, Cambridge, UK), anti-BACE (1:1000, sc-33711, Santa Cruz Biotechnology, Dallas, TX, USA), anti-GFAP (1:1000, ab53554, Abcam), anti-Iba1 (1:1000, ab15690, Abcam), or anti-β-actin (1:3000, #3700, Cell Signaling, Danvers, MA, USA). After overnight incubation, membranes were washed thoroughly in PBS-T buffer and incubated for 1 h with the corresponding secondary antibody diluted at 1:3000. The membranes were then washed, and immunoreactivity was detected using an enhanced chemiluminescence (ECL) kit (NEL105001EA, Perkin Elmer, Waltham, MA, USA).

### 4.5. Positron Emission Tomography (PET) Scans

[^18^F]FDG was provided by the Radiopharmaceutical Production Team at KIRAMS. The radiochemical purity at the end of the synthesis was >95%. PET experiments were conducted using an animal-dedicated PET scanner (NanoScan^®^, Mediso Medical Imaging Systems, Budapest, Hungary). The scanner has a peak absolute system sensitivity >9% in the 250–750 keV energy window, an axial field of view of 28 cm, a transaxial field of view of 35–120 mm and a transaxial resolution of 0.7 mm at 1 cm off center.

All mice were anesthetized with 2.5% isoflurane in oxygen and 8.2 ± 0.5 MBq of [^18^F]FDG was administered via the tail vein. PET scan was performed 40–60 min post-injection (p.i.). The image scans were acquired with an energy window of 400–600 keV. All images were reconstructed using the three-dimensional ordered subset expectation maximization (3D-OSEM) algorithm with four iterations and six subsets. For attenuation correction and anatomical reference, micro-CT imaging was conducted immediately after PET using an X-ray voltage of 50 kVp at 0.16 mA.

Three-dimensional VOIs were drawn to compare regional brain PET uptake between groups. Individual PET images were spatially normalized to the M. Mirrione T2-weighted mouse brain MRI template embedded in the PMOD software (version 3.7, PMOD Group, Graubünden, Switzerland). The obtained uptake value, represented as the SUV, was determined for each region. SUV values were calculated to normalize the differences in the injected dose and body weight. The cortex, striatum, hippocampus, thalamus, amygdala, and cerebellum were selected as volumes of interest (VOIs).

### 4.6. Statistical Analysis

Data are expressed as mean ± standard deviation (SD). One-way ANOVA followed by the Tukey post hoc analysis or Student’s unpaired two-tailed *t*-test were performed using the GraphPad Prism program (version 8.0, GraphPad Software, San Diego, CA, USA) and indicated in the figure legends. Values with *p* < 0.05 were considered statistically significant.

## Figures and Tables

**Table 1:** Comparison of the regional SUV values.

Group		Brain Uptake Value (SUV)					
	Cortex	Striatum	Hippocampus	Thalamus	Hypothalamus	Amygdala	Cerebellum
WT	1.56 ± 0.13	1.87 ± 0.16	1.96 ± 0.20	1.97 ± 0.16	1.57 ± 0.12	1.15 ± 0.09	2.18 ± 0.26
5xFAD + Veh	1.35 ± 0.12	1.65 ± 0.13	1.65 ± 0.17	1.80 ± 0.14	1.42 ± 0.08	1.05 ± 0.09	1.92 ± 0.13
5xFAD + Ari	1.61 ± 0.05	1.97 * ± 0.08	2.07 ** ± 0.08	2.17 * ± 0.10	1.67 ± 0.07	1.32 ± 0.05	2.09 ± 0.12

Data are presented as the mean ± the SEM (*n* = 5 for WT, *n* = 6 for 5xFAD + Veh, *n* = 8 for 5xFAD + Ari). Statistical significance using Student’s *t*-test was defined as a *p* value less than 0.05 for the comparisons between 5xFAD + Veh and 5xFAD + Ari groups (* *p* < 0.05, ** *p* < 0.01).

## Data Availability

The data that support the findings of this study are available from the corresponding author upon reasonable request.
